# RT-qPCR and split-luciferase assays enable batch standardization and analysis of engineered virus-like particle transduction

**DOI:** 10.1016/j.omta.2026.201685

**Published:** 2026-02-09

**Authors:** Lucia Nicosia, Joss B. Murray, Emma Collins, Lisa Lonetti, Patrick T. Harrison, Martina F. Scallan

**Affiliations:** 1 Department of Physiology, University College Cork, T12 YT20 Cork, Ireland; 2 School of Microbiology, University College Cork, T12 K8AF Cork, Ireland; 3 Division of Pulmonary Medicine, Cincinnati Children’s Hospital, Cincinnati, OH 45228, USA

**Keywords:** virus-like particles, reporter cell line, luminescence, adenine base editing, delivery

## Abstract

Recently developed engineered virus-like particles (eVLPs) have emerged as a promising delivery vehicle for ribonucleoprotein gene editing complexes. Variability in eVLP batch production may, however, hinder reproducibility and standardization across pre-clinical investigations designed to characterize and optimize this platform. Ultimately, stringent production, purification, and quantification processes will be required for this technology to reach the clinic. In this study, we developed two titration methods for base editor (BE)-eVLPs: a spacer-agnostic quantitative reverse-transcription PCR (RT-qPCR) assay to quantify the copy number of sgRNA molecules, and a NanoBiT luciferase-based approach to estimate vesicular stomatitis virus envelope glycoprotein (VSV-G) abundance, per μL of BE-eVLP preparation. We further engineered an LgBiT-expressing reporter cell line to monitor BE-eVLP transduction kinetics in real time. Our findings reveal that both RT-qPCR and HiBiT-based quantification enable effective batch-to-batch standardization of BE-eVLP preparations. Further, the LgBiT-expressing reporter cell line was effective in real-time monitoring of transduction kinetics. BE-eVLP transduction was proven dependent on endosomal acidification and was constrained by cellular endocytic capacity. Paired with accurate quantification of BE-eVLP preparations and using HiBiT-tagged virus-surface glycoproteins, the LgBiT reporter cell line can facilitate comparison of transduction and levels of editing achieved across emergent eVLP platforms and pseudotypes.

## Graphical abstract

**Figure:**
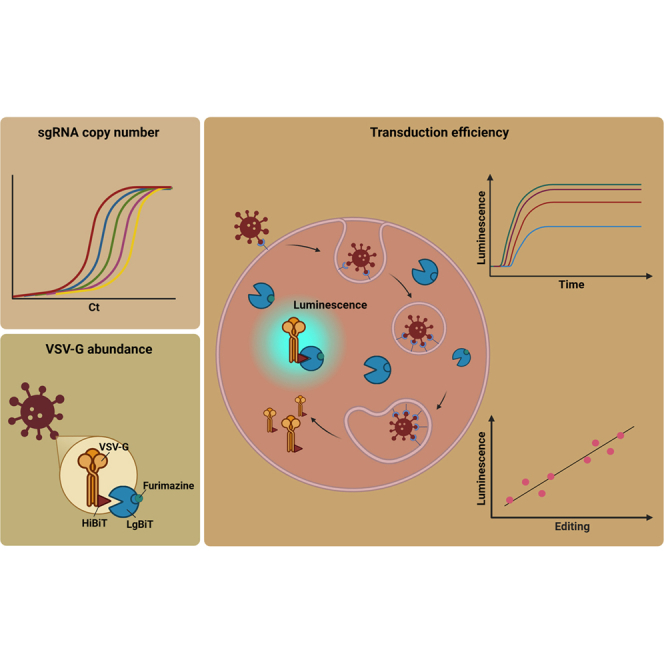

## Teaser

Nicosia and colleagues present two quantification methods for base editor-engineered virus-like particles (BE-eVLPs) and develop a reporter system to monitor transduction in real time.

## Introduction

Engineered virus-like particles (eVLPs) have recently emerged as a promising delivery platform for CRISPR-based applications both *in vitro* and *in vivo*.^1^^,^^2^^,^^3^ We and others have used base editor (BE) eVLPs (BE-eVLPs) for the delivery of ribonucleoprotein complexes (RNPs) of BE and single guide RNA (sgRNA), encapsulated within Moloney murine leukemia virus (MMLV)-enveloped capsids, pseudotyped with vesicular stomatitis virus envelope glycoprotein (VSV-G).^1^^,^^3^^,^^4^^,^^5^^,^^6^

Reliable quantification of eVLP preparations is critical for in-laboratory and between-laboratory experimental reproducibility, for fundamental investigations into cellular responses to eVLPs, as well as to inform translation toward clinical applications. Titration by volume fails to account for batch-to-batch variation in particle composition. Enzyme-linked immunosorbent assays (ELISAs) measuring p30 (MLV gag-core antigen) and Cas9 protein concentrations are the current standard,^1^^,^^2^^,^^3^^,^^7^ but are both time-consuming and costly.

Here, we describe the development of two alternative titration methods for BE-eVLP preparations and the generation of a reporter cell line to study eVLP transduction dynamics.

We propose (1) a spacer-agnostic quantitative reverse-transcription PCR (RT-qPCR) approach for the estimation of sgRNA copies per μL of BE-eVLP preparation, and (2) a NanoBiT luciferase-based assay for the quantification of VSV-G abundance per μL of preparation. We implemented these titration strategies for their ability to provide functionally relevant information about BE-eVLP preparations. The RT-qPCR approach enables the quantification of “active” BE:sgRNA RNPs by detecting sgRNA molecules, while the NanoLuc luciferase-based assay measures VSV-G content, which reflects transduction competence. VSV-G incorporation and glycosylation status are, in fact, crucial determinants of VLP entry efficiency,^3^^,^^8^^,^^9^ making envelope glycoprotein quantification suitable for predicting eVLP batch transduction capacity. To investigate cellular uptake dynamics with HiBiT-tagged BE-eVLPs, we developed an LgBiT luciferase-based reporter cell line enabling real-time monitoring of BE-eVLP transduction events via luminescence. In the same cell line, we also compared the efficacy of the proposed methods of titration in minimizing variability across experiments performed with independent batches of BE-eVLP preparations.

## Results

### Spacer-agnostic RT-qPCR assay for the titration of BE-eVLP preparations based on sgRNA copy number

**Figure 1:**
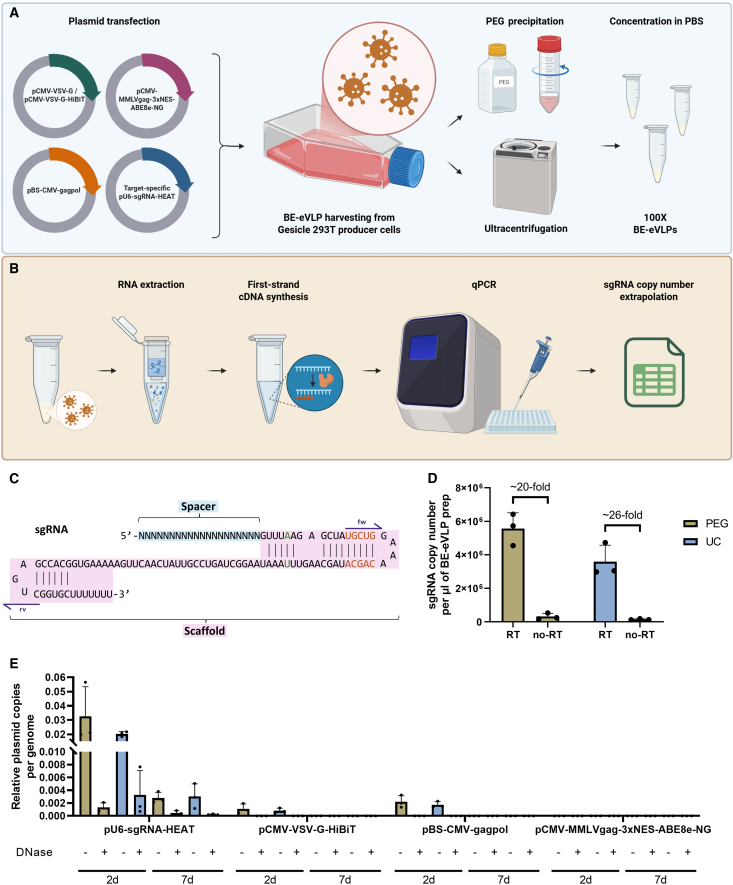
Spacer-agnostic RT-qPCR assay for the titration of BE-eVLP preparations based on sgRNA copy number (A) Representative schematic of BE-eVLP production and purification. Gesicle producer cells are co-transfected with plasmids expressing (1) MMLV gag-pol proteins; (2) VSV-G envelope glycoprotein, fused to a HiBiT tag for specific experiments; (3) NG-ABE8e; and (4) sgRNA. The culture supernatant is clarified and BE-eVLPs concentrated via PEG precipitation or ultracentrifugation. (B) Representative schematic of BE-eVLP processing for the RT-qPCR assay. RNA is extracted from purified BE-eVLPs and reverse-transcribed with random hexamers. qPCR is performed on the BE-eVLP cDNA to quantify the copy number of sgRNAs per μL of BE-eVLP preparation. (C) Schematic of the sgRNA structure, with spacer highlighted in blue, scaffold in pink, HEAT modifications colored in green and orange, and scaffold-specific qPCR primers in purple. (D) Copy number of sgRNAs from three independent BE-eVLP preparations, which were either PEG precipitated or ultracentrifuged. *n* = 3; bars represent mean ± SD. (E) Relative plasmid copies per genome of LgBiT-HEK293 cells at day 2 or 7 post-transduction with three independent PEG precipitated or ultracentrifuged BE-eVLP preparations, with or without whole-prep DNase treatment. *n* = 3; bars represent mean ± SD.

The BE-eVLPs used in this study were produced by co-transfecting producer cells with plasmids (Figure 1A) expressing (1) MMLV gag (truncated) and pol proteins, (2) VSV-G (or HiBiT-tagged VSV-G), (3) the adenine BE ABE8e-NG^10^ fused with MMLV gag protein, and (4) an sgRNA targeting either HEK293 cell genomic site 2 or site 3, hereafter referred to as HEK2 or HEK3, or exon 11 of the *CFTR* gene (CFex11).^11^ While HEK2 and HEK3 sgRNAs bind standard benchmark loci for characterizing BEs’ activity,^12^ the CFex11 sgRNA was selected to study editing events in a more complex chromatin context.^11^^,^^13^ The particles were concentrated either with a polyethylene glycol (PEG) solution or by ultracentrifugation (Figure 1A).

To estimate the number of sgRNA copies per μL of independent BE-eVLP batches, we designed an RT-qPCR assay (Figure 1B) with primers targeting the sgRNA scaffold sequence (Figure 1C, highlighted in pink)—the invariant portion of the sgRNA that mediates binding to the editor. The scaffold used in this study also incorporated the commonly used hybridization extended A-T inversion (HEAT) modifications, as described in previous work (Figure 1C, in green and orange).^14^^,^^15^ This layout allows the RT-qPCR assay outlined here to be applicable to any sgRNA independent of the spacer (Figure 1C, highlighted in blue), which is the target-specific region that hybridizes to the complementary DNA sequence.^14^^,^^15^^,^^16^

We first generated a standard curve using a chemically synthesized DNA oligonucleotide corresponding to the cDNA sequence of each sgRNA (including both spacer and scaffold). qPCR amplification of serially diluted standards yielded efficiencies between ∼92% and ∼98%, with R^2^ values exceeding 0.998, and a single amplicon detected by melt-curve analysis (Figure S1A), confirming assay linearity, absence of saturation, and absence of primer dimers.^17^

We then quantified the copy number of sgRNAs per μL of each BE-eVLP preparation on 10- or 100-fold dilutions of first-strand cDNA samples from BE-eVLP RNA extracts. We calculated the log_10_ of the concentration (in ng) of each test sample from its Ct value by applying Equation 1. We then extrapolated the number of sgRNA copies per μL of qPCR reaction by applying Equation 2.^18^ Every step of the RT-qPCR analysis is described in the materials and methods section and reported in a supplementary Excel spreadsheet (Table S1) that can be used as a template.

(Equation 1) y=mx+b.

Equation 1 gives the slope-intercept form of the standard curve.

(Equation 2) $$\text{Number}\;\text{of}\;\text{copies}=\frac{\text{ng}\;\text{of}\;\text{test}\;\text{sample}\times 6.022\times 10^{23}\frac{\text{molecules}}{\text{mol}}}{\text{amplicon}\;\text{length}\;\left(\text{bp}\right)\times 330\frac{g}{\text{mol}}\times 1\times 10^{9}\;\frac{\text{ng}}{g}}\text{.}$$

Equation 2 is the formula for the extrapolation of the sgRNA copy number.

Figure 1D shows average sgRNA copy number per μL from three independent BE-eVLP preparations carrying HEK2, HEK3, or CFex11 sgRNA, which were either PEG precipitated (∼6 × 10^6^) or ultracentrifuged (∼4 × 10^6^), concentrated 100-fold, and DNase treated during column-based RNA extraction. While substantially lower (∼20- to 26-fold), the no-RT control of all preparations produced a detectable signal (Figure 1D). An important limitation of RT-qPCR for the quantification of sgRNAs is the potential for any primer pair to detect not only sgRNA molecules but also residual sgRNA-encoding plasmid. To confirm plasmid carryover in BE-eVLPs, we also performed a PCR with primers amplifying the ampicillin resistance (Amp^R^) cassette, which is present in every plasmid used for particle production. Gel electrophoresis showed amplification of the Amp^R^ region (427-bp amplicon) in both cDNA and no-RT controls from the DNase-untreated RNA extract of a PEG precipitated preparation (Figure S1B). To determine whether the amplicon originated from a complete plasmid or residual fragments, and to identify which plasmid(s) could be carried over in the BE-eVLP preparations, we next transformed the cDNA and no-RT control samples into competent *E. coli* cells. The no-RT control yielded a colony; the plasmid DNA purified from this colony was sequenced and showed 100% identity with the complete sgRNA-encoding plasmid used in the production of the BE-eVLP preparation. The growth of just one colony does not exclude the possibility that other plasmids, perhaps less abundant, may be present as well.

These findings raised concerns over the possibility that any plasmid carried over in the BE-eVLPs may be co-delivered into target cells upon transduction. To investigate this, we performed a qPCR on DNA extracted from human embryonic kidney 293 (HEK293) cells transduced with equal volumes of three independent BE-eVLP preparations (targeting HEK2, HEK3, or CFex11). The particles were either PEG precipitated or ultracentrifuged, and DNA was extracted from transduced cells at day 2 or 7 post-transduction. We also evaluated the effect of DNase treatment on whole preparations prior to transduction (Figure 1E). For this assay, a standard curve was built with serial dilutions of each plasmid and primers were included for the ribosomal protein lateral stalk subunit P0 (*RPLP0*) gene as an internal control to normalize against genome copy number across all conditions. The BE-encoding plasmid was not detectable under any condition, while VSV-G and gag-pol plasmids showed only trace amounts at day 2 post-transduction (Figure 1E). In contrast, the sgRNA plasmid was detected in every condition, although at negligible levels (<6 copies per 100 genomes), which decreased over time (Figure 1E) suggesting no genomic integration. While direct DNase treatment of harvested BE-eVLP preparations reduced plasmid contamination (Figure 1E), this approach likely resulted in substantial reduction of BE-eVLP titer, as discussed in subsequent sections. We further evaluated BE-eVLP-dose-dependent plasmid contamination by examining DNA extracts from biological triplicates of ∼30,000 cells, transduced with 1 or 5 μL of a BE-eVLP preparation. At 72 h post-transduction, 0.1 and 0.5 ng of sgRNA-encoding plasmid were detected, respectively, in cells transduced with 1 and 5 μL of BE-eVLPs (a 5-fold change; Figure S1C).

### HiBiT assay for titration of BE-eVLP preparations based on VSV-G abundance

**Figure 2:**
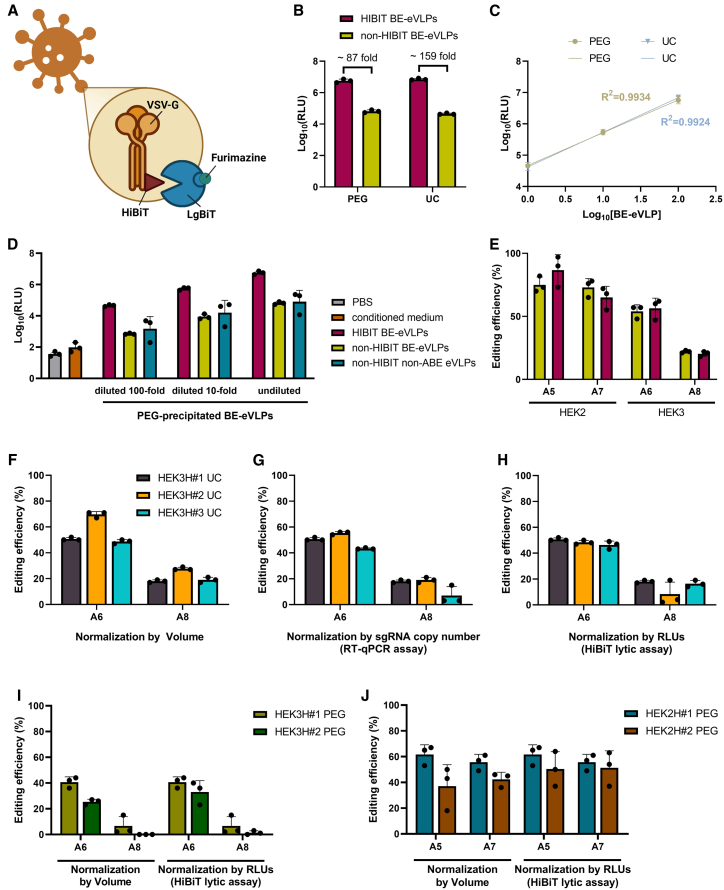
HiBiT assay for titration of BE-eVLP preparations based on VSV-G abundance (A) Representative schematic of HiBiT-tagged VSV-G interacting with LgBiT and furimazine. (B) Luminescence readings (log_10_ RLUs) of three independent HiBiT-tagged BE-eVLP preparations and three independent non-HiBiT-tagged BE-eVLP preparations, either PEG precipitated or ultracentrifuged. *n* = 3; bars represent mean ± SD. (C) Correlation between luminescence readings (log_10_ RLUs) and dilutions of three independent HiBiT-tagged BE-eVLP preparations (undiluted, diluted 10-fold, and diluted 100-fold), either PEG precipitated or ultracentrifuged. *n* = 3; bars represent mean ± SD. (D) Luminescence readings (log_10_ RLUs) of PBS, conditioned medium from producer cells (corresponding to 1 μL of a 100-fold diluted BE-eVLP preparation), and three independent PEG precipitated HiBiT-tagged, non-HiBiT, non-HiBiT non-ABE eVLP preparations (undiluted, diluted 10-fold, and diluted 100-fold). *n* = 3; bars represent mean ± SD. (E) Editing efficiency (%) analyzed with EditR software at positions A5 and A7 of the HEK2 protospacer and A6 and A8 of the HEK3 protospacer, using PEG precipitated BE-eVLP preparations normalized by RT-qPCR; *n* = 3; bars represent mean ± SD. (F–H) Editing efficiency (%) at positions A6 and A8 of the HEK3 protospacer using equal volumes of three independent ultracentrifuged HiBiT-BE-eVLP batches (F) or adjusted volumes of the same preparations normalized, as described in the text, via RT-qPCR (G) or HiBiT assay (H). *n* = 3; bars represent mean ± SD. (I and J) Editing efficiency (%) at positions A6 and A8 of the HEK3 protospacer (I) or at A5 and A7 of the HEK2 protospacer (J), using equal volumes of PEG precipitated HiBiT-BE-eVLP preparations or adjusted volumes of the same preparations normalized via HiBiT assay. *n* = 3; bars represent mean ± SD.

Luciferase reporter systems are characterized by high and broad-range sensitivity^19^ and a more rapid and cost-effective workflow compared to antibody-based assays such as ELISA.^19^ Building on previous work that engineered complementing subunits of a split NanoBiT luciferase^20^ to measure protein interaction dynamics,^21^ we tested the applicability of the split HiBiT/LgBiT system^22^ for the quantification of glycoprotein VSV-G abundance in BE-eVLP preparations (Figure 2). HiBiT is an 11-amino acid peptide (1.3 kDa) that binds with high affinity (Kd = 700 pM) to a larger complementary subunit called LgBiT (18 kDa). The association of HiBiT and LgBiT reconstitutes the NanoBiT luciferase enzyme, which, upon substrate addition, produces a luminescent signal (Figure 2A).^22^

To test this system for particle titration, we produced new BE-eVLP batches by replacing the plasmid encoding VSV-G with one expressing a VSV-G fusion protein tagged with HiBiT at the C terminus.^23^ The resulting BE-eVLPs were quantified using the Nano-Glo HiBiT Lytic Detection System, which provides the complementary LgBiT protein necessary for luciferase reconstitution with the HiBiT tag, and the furimazine substrate for luminescence emission in solution.

Independent BE-eVLP preparations pseudotyped with HiBiT-tagged VSV-G (herein referred to as HiBiT BE-eVLPs) reported similar levels of VSV-G abundance when PEG precipitated or ultracentrifuged (Figure 2B) and exhibited a linear dose-response (Figure 2C). Unexpectedly, BE-eVLPs pseudotyped with VSV-G lacking the HiBiT tag (non-HiBiT BE-eVLPs) generated a signal above background levels (Figure 2D), albeit ∼120-fold lower than HiBiT BE-eVLPs on average (Figure 2B).

Given that the no-eVLP controls (PBS and conditioned medium from non-transfected producer cells) produced only a negligible signal (Figure 2D), we speculate that the luminescence generated by non-HiBiT BE-eVLPs is unlikely a result of spontaneous complementation. Instead, the non-specific signal may be due to cross-reactivity between LgBiT and peptides within the BE-eVLPs that share similar amino acid sequence or properties with HiBiT. To investigate this, we computationally searched for HiBiT-like sequences (VSGWRLFKKIS) across VSV-G, gag-pol, and BE plasmids. DNA sequences were translated in all six reading frames and analyzed using exact sequence matching, fuzzy matching based on Hamming distance across 11-amino acid windows, and property-based matching grouping residues by their biochemical characteristics (e.g., hydrophobic, polar, and charged). Top candidates per plasmid are reported in Tables S2 and S3 and annotated in Figure S2. We identified potential HiBiT-like motifs in each plasmid-encoded BE-eVLP protein component, with some sequences appearing in more than one plasmid. To determine which peptide may be contributing to the non-specific luminescent signal, we produced eVLPs pseudotyped with non-tagged VSV-G and lacking the BE, reasoning that gag-pol (essential for assembly) and VSV-G (essential for transduction) could not be eliminated. Non-HiBiT-non-ABE eVLPs showed similar luminescence levels in the lytic assay to non-HiBiT BE-eVLPs (Figure 2D), indicating ABE8e-NG does not contribute to luminescence, and implicating VSV-G or gag-pol as the source.

Next, we evaluated whether incorporating the HiBiT tag would affect transduction and, by extension, editing efficiency. Sanger sequencing on DNA extracts from transduced cells revealed no significant difference in editing efficiency between HiBiT- and non-HiBiT BE-eVLPs (Figure 2E).

**Figure 3:**
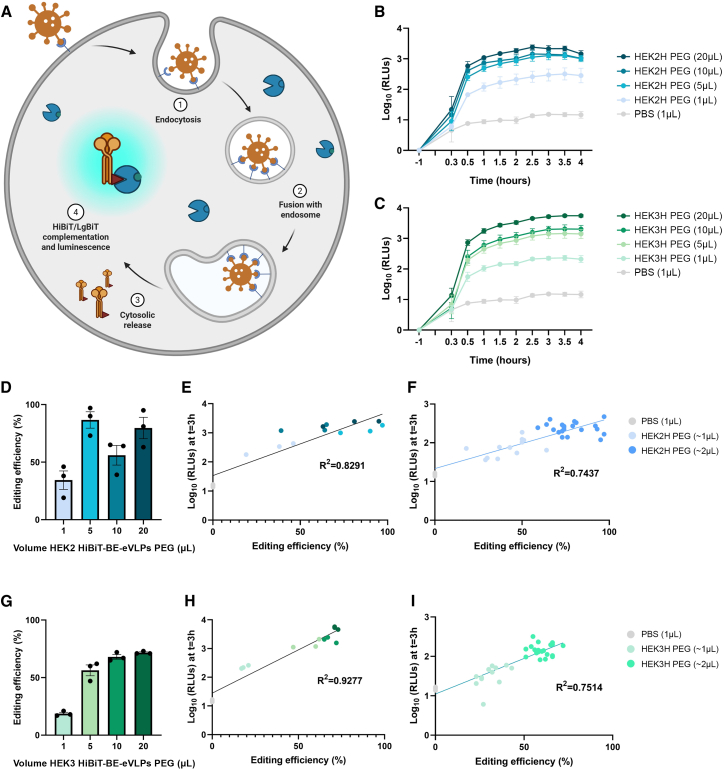
LgBiT assay for real-time monitoring of BE-eVLP transduction of reporter cell line (A) Representative schematic of LgBiT reporter cell line transduced by HiBiT-tagged BE-eVLPs. The BE-eVLPs are endocytosed and their components that escape lysosomal degradation are released into the cell cytoplasm, where the HiBiT tag on VSV-G can complement with LgBiT and, provided with furimazine, generate luminescence. (B) Luminescence readings (log_10_ RLUs) of PBS and increasing doses of a HEK2-targeting HiBiT BE-eVLP preparation (HEK2H) over a period of 4 h in the LgBiT reporter cell line. *n* = 3; bars represent mean ± SD. (C) Luminescence readings (log_10_ RLUs) of PBS and increasing doses of an HEK3-targeting HiBiT BE-eVLP preparation (HEK3H) over a period of 4 h in the LgBiT reporter cell line. *n* = 3; bars represent mean ± SD. (D) Editing efficiency (%) analyzed with EditR software at position A5 of the HEK2 protospacer, with increasing doses of BE-eVLPs (∼34.3% with 1 μL, ∼86.7% with 5 μL, ∼56% with 10 μL, ∼79.7% with 20 μL). *n* = 3; bars represent mean ± SEM. These data were retrieved from the same monolayers of cells analyzed in (B). (E) Correlation between luminescence readings (log_10_ RLUs, B) and editing efficiency (%, D) at A5 with increasing doses of HEK2-targeting HiBiT-tagged BE-eVLPs at time t = 3 h. *n* = 3 per dose. (F) Correlation between luminescence readings (log_10_ RLUs) and editing efficiency (%) at A5 with HEK2-targeting HiBiT-tagged BE-eVLPs in the range of ∼1 and ∼2 μL at time t = 3 h. (G) Editing efficiency (%) analyzed with EditR software at position A6 of the HEK3 protospacer, with increasing doses of BE-eVLPs (∼18.7% with 1 μL, ∼56.3% with 5 μL, ∼68% with 10 μL, ∼71.7% with 20 μL). *n* = 3; bars represent mean ± SEM. These data were retrieved from the same monolayers of cells analyzed in (C). (H) Correlation between luminescence readings (log_10_ RLUs, C) and editing efficiency (%, G) at A6 with increasing doses of HEK3-targeting HiBiT-tagged BE-eVLPs at time t = 3 h. *n* = 3 per dose. (I) Correlation between luminescence readings (log_10_ RLUs) and editing efficiency (%) at A6 with HEK3-targeting HiBiT-tagged BE-eVLPs in the range of ∼1 and ∼2 μL at time t = 3 h.

Finally, we tested whether the proposed titration methods could standardize editing efficiencies across individual BE-eVLP batches. For each target locus (HEK2, HEK3, and *CFTR*), independent preparations were normalized by matching (1) volume (Figure 2F), (2) sgRNA copy number via RT-qPCR quantification (e.g., adjusting volume input of Prep #2 and #3 to match the number of sgRNA copies of Prep #1, Figure 2G), or (3) relative light units (RLUs) via HiBiT-based quantification (e.g., adjusting volume input to match the RLUs of Prep #1, Figure 2H). Editing efficiency was assessed at two adenines within the editing window for HEK2 (A5 and A7), and HEK3 (A6 and A8) (Figures 2F–2J); no editing was detectable via Sanger sequencing at *CFTR* exon 11 (not shown). Both the RT-qPCR and the HiBiT assays outperformed volume-based normalization across ultracentrifuged BE-eVLP preparations, with HiBiT-based normalization achieving the closest alignment to the reference preparation (Figures 2F–2H). Since PEG precipitation can co-precipitate soluble proteins along with eVLPs, free HiBiT-tagged VSV-G (not eVLP-associated) could theoretically contribute to luminescence signal in the lytic assay and confound HiBiT-based quantification. To address this potential concern, we validated HiBiT-based normalization also using PEG precipitated preparations, which, to further increase batch titer variability, were deliberately prepared at different concentrations. Average editing efficiencies achieved across batches (Figures 2I and 2J) suggest that HiBiT-based standardization also applies to PEG precipitated BE-eVLPs.

### LgBiT assay for real-time monitoring of BE-eVLP transduction of reporter cell line

Next, we generated a HEK293 reporter cell line stably expressing the HiBiT-complementary LgBiT subunit (Figure 3A), which would enable real-time monitoring of transductions performed with HiBiT-tagged BE-eVLPs. To achieve stable LgBiT expression, a LgBiT-puromycin expression cassette was integrated into the AAVS1 safe-harbor locus of HEK293 cells via CRISPR/Cas9-mediated homology-directed repair (HDR). Following puromycin selection, clonal cell populations carrying the integrated cassette were established (Figure S3). Upon transduction of reporter LgBiT-HEK293 cells with HiBiT-BE-eVLPs, the HiBiT tag, fused to the C terminus of the VSV-G, can complement with intracellular LgBiT following successful cytoplasmic release. Concurrent addition of Vivazine (a version of furimazine suitable for live cell assays) enables sensitive real-time detection of HiBiT-LgBiT complementation events through luminescence (Figure 3A).

As an initial test to verify that the assay would yield a measurable luminescent signal, we transduced reporter LgBiT cells with 1 μL of HiBiT-BE-eVLPs targeting HEK2 or HEK3 (HEK2H and HEK3H BE-eVLPs), and we monitored luminescence every 30 min over a 4-h period (Figures 3B and 3C). To account for well-to-well variability, per-well normalization was applied, by dividing the luminescence values at each time point by the corresponding RLUs pre-treatment (measured prior to the experiment, at time t = −1 h). Control monolayers receiving PBS instead of HiBiT-BE-eVLPs exhibited negligible luminescence throughout (Figures 3B and 3C). In contrast, monolayers transduced with HiBiT-BE-eVLPs exhibited luminescence following addition of particles and Vivazine (time 0), with a sharp increase over the first 30 min. Signal intensity peaked at approximately t = 2.5–3 h and subsequently plateaued (Figures 3B and 3C).

The possibility that the plateau might reflect limited availability of substrate was considered, specifically that Vivazine levels had reached a steady state around 3 h, where substrate consumption is balanced by degradation. In this scenario, additional HiBiT:LgBiT complex formation might not yield proportionally increased luminescence due to insufficient Vivazine. A similar limitation could apply to intracellular LgBiT availability: once the majority of LgBiT molecules are bound by HiBiT, further complementation would not occur. To address this, a 5-fold higher input volume (5 μL) of HiBiT-BE-eVLPs was tested. The magnitude of emitted luminescence increased proportionately (∼5-fold higher than that observed with 1 μL; Figures 3B and 3C). This indicates that the plateauing of luminescence readings over time is not due to limitation of substrate availability or availability of LgBiT, but more likely represents the full extent of delivery of eVLP components out of the endosome for each condition. Doubling of eVLP dose to 10 μL and then to 20 μL did not result in further proportional increases in emitted luminescence. However, the same pattern of emission of luminescence was observed across all tested conditions, consistently plateauing at time t = ∼2.5–3 h. These observations suggest that the level at which luminescence readings plateau is indicative of constraints in transduction, rather than substrate or LgBiT availability. At lower doses of eVLPs, the dose may be limiting; at higher doses of eVLPs, the capacity of the cell to deliver the eVLPs becomes saturated.

To explore this further, editing efficiency was assessed for each condition. Comparable levels of editing were achieved with 5, 10, and 20 μL of either HEK2H or HEK3H BE-eVLPs (Figures 3D and 3G), which indicated a saturation effect in editing at higher input volumes. When editing efficiency (%) was plotted against luminescence values (RLUs), a strong correlation was found (R^2^ = 0.8291 and 0.9277; Figures 3E and 3H), which highlighted that the similar luminescence levels observed across the 5, 10, and 20 μL doses of HBiT-BE-eVLPs are indeed associated with comparable editing efficiencies (Figures 3E and 3H).

Transductions with lower input volumes (∼1–2 μL for both HEK2 and HEK3 targets) instead showed increased levels of editing with increased dose. The strength of the correlation between luminescence (RLUs) and editing efficiency was reduced from R^2^ = 0.8291 to R^2^ = 0.7437 for HEK2 and from R^2^ = 0.9277 to R^2^ = 0.7514 for HEK3 (Figures 3F and 3I), possibly due to limited sensitivity of the luminescence readout at lower input levels. However, transducing the cells with two sequential 1 μL doses of HiBiT-BE-eVLPs, one at time t = 0 h and another at t = ∼3 h, did not result in significant changes in luminescence (Figure S4A) or editing efficiency (Figure S4B) relative to a single 1 μL dose. These observations are consistent with the possibility that the first dose initiates cell intrinsic antiviral responses that reduce endocytic capacity, thus constraining transduction.

### Validation of the reporter cell line and transduction dynamics

**Figure 4:**
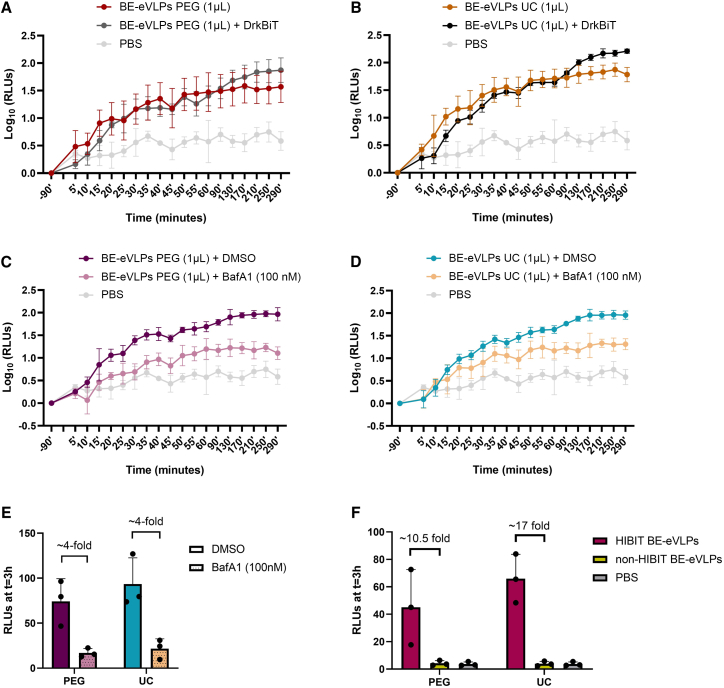
Validation of the reporter cell line and transduction dynamics (A) Luminescence readings (log_10_ RLUs) with PBS or 1 μL of three independent PEG precipitated HiBiT-BE-eVLP preparations, with or without DrkBiT, over a period of ∼5 h, with 5-min intervals in the first hour. *n* = 3; bars represent mean ± SD. (B) Luminescence readings (log_10_ RLUs) with PBS or 1 μL of three independent ultracentrifuged HiBiT-BE-eVLP preparations, with or without DrkBiT, over a period of ∼5 h, with 5-min intervals in the first hour. *n* = 3; bars represent mean ± SD. (C) Luminescence readings (log_10_ RLUs) with PBS or 1 μL of three independent PEG precipitated HiBiT-BE-eVLP preparations, with or without BafA1 (100 nM) treatment, over a period of ∼5 h, with 5-min intervals in the first hour. *n* = 3 (*n* = 2 for DMSO); bars represent mean ± SD. (D) Luminescence readings (log_10_ RLUs) with PBS or 1 μL of three independent ultracentrifuged HiBiT-BE-eVLP preparations, with or without BafA1 (100 nM) treatment, over a period of ∼5 h, with 5-min intervals in the first hour. *n* = 3; bars represent mean ± SD. (E) Luminescence readings (RLUs) at time t = 3 h post-transduction with three independent BE-eVLP preparations that were either PEG precipitated or ultracentrifuged (from C and D), with or without BafA1 (100 nM) treatment. *n* = 3; bars represent mean ± SD. (F) Luminescence readings (RLUs) at time t = 3 h post-transduction with three independent HiBiT- and non-HiBiT BE-eVLP preparations, which were either PEG precipitated or ultracentrifuged, compared to baseline (PBS). *n* = 3; bars represent mean ± SD.

To validate that the observed luminescence plateau reflected intracellular events, we addressed a potential confound: LgBiT can leak from recipient cells into the medium,^23^^,^^24^ where it could interact with HiBiT-tagged VSV-G proteins (either on BE-eVLPs or as free protein) and generate extracellular luminescent signals that are not specific to transduction. We, therefore, repeated luminescence measurements with 1 μL of BE-eVLPs in the presence or absence of DrkBiT, an inhibitory peptide that competitively binds extracellular LgBiT and quenches extracellular luminescence.^23^^,^^24^ We compared three independent eVLP preparations that were either PEG precipitated (Figure 4A) or ultracentrifuged (Figure 4B), anticipating that PEG preparations might contain more free HiBiT-VSV-G protein that could increase background signal. Under our experimental conditions, luminescence levels were similar between DrkBiT-treated and untreated cells, regardless of eVLP concentration method, implying that the luminescence signals detected with this assay derived from intracellular complementation.

Next, we tested whether luminescence specifically reflected transduction by treating cells with 100 nM bafilomycin A1 (BafA1), a V-ATPase inhibitor that prevents endosomal acidification and blocks VSV-G-mediated membrane fusion.^25^^,^^26^^,^^27^ BafA1 treatment reduced luminescence by ∼4-fold (Figures 4C–4E) confirming that the signal depends on endosomal acidification-dependent transduction. Measuring luminescence every 5 min during the first hour and up to ∼5 h also revealed that BE-eVLP transduction follows sigmoidal kinetics (Figures 4A–4D).

As a final control, we tested whether non-HiBiT BE-eVLPs could generate a non-specific signal in the reporter cell line. While the lytic HiBiT assay had recorded above-background luminescence from these particles (Figure 2B), >10-fold lower luminescence was detected in LgBiT HEK293 cells transduced with non-HiBiT-BE-eVLPs relative to HiBiT-BE-eVLPs (Figure 4F). In fact, luminescence readings approached background levels (Figure 4F), validating the specificity of the *in vitro* LgBiT assay for HiBiT-BE-eVLPs.

## Discussion

Accurate quantification of BE-eVLP preparations is key for experimental design, standardization across batches, and data interpretation. To this end, we established two BE-eVLP titration methods and a reporter cell line enabling real-time monitoring of transduction dynamics and comparison of particle dosing with editing efficiencies.

First, we developed an RT-qPCR assay that quantifies the copy number of sgRNAs per μL of BE-eVLP preparation. A programmed Excel template is provided in the supplemental information (Table S1) to support analysis of data generated using our RT-qPCR design. Optimized reaction conditions and primer sequences targeting the sgRNA scaffold region are set out, making this assay readily and broadly applicable to any sgRNA, independent of the editing target sequence. Banskota et al. calculated an abundance of 50 BE molecules per BE-eVLP v.v4.^1^ Assuming one copy of sgRNA/BE molecule, a titer of BE-eVLPs/μL can be deduced, enabling the initiation of transduction at a known multiplicity of transduction (ratio of BE-eVLPs to cells).^4^

Importantly, the RT-qPCR assay described in this study revealed traces of plasmid DNA carryover from BE-eVLPs into transduced cells. The sgRNA-encoding plasmid was the most frequently carried over, likely due to its smaller size or higher copy number relative to other co-transfected plasmids. While co-delivered sgRNA plasmids could theoretically be transcribed by host cells and reload the BE for continued editing, this scenario would be limited to the first ∼24 h post-transduction, as the BE-encoding plasmid is not carried over with BE-eVLPs and previous studies have demonstrated a ∼24-h half-life for Cas9 protein.^28^^,^^29^

DNase I treatment of whole BE-eVLP preparations substantially reduced plasmid DNA contamination. However, luminescence readings and Sanger sequencing from cells transduced with DNase-treated BE-eVLPs demonstrated reduced levels of transduction (Figure S5). This reduction likely reflects loss of active particles during the DNase inactivation and pelleting steps. In the context of *in vivo* and future clinical applications, the eVLP production process could be enhanced by engineering a producer cell line that stably expresses at least the sgRNA and possibly also the other VLP components. This would eliminate the requirement for transient plasmid transfection entirely. Knockout of the low-density lipoprotein receptor in an eVLP-producer cell line would prevent self-transduction from VSV-G-pseudotyped eVLPs. Such approaches could enhance both the purity and scalability of eVLP production.

The second quantification method we developed—the HiBiT-based assay—measured HiBiT-tagged VSV-G abundance in HiBiT BE-eVLPs by leveraging a split NanoBiT luciferase system. Compared to immune-based approaches like ELISA, this method offers a faster and more cost-effective alternative for BE-eVLP titration and is designed for batch-to-batch normalization by relative quantification. When BE-eVLP dose was normalized by HiBiT signal prior to transduction, we observed highly reproducible editing efficiencies across independent batches, validating this approach as an effective strategy for experimental standardization.

The combined use of HiBiT-tagged BE-eVLPs and an LgBiT-expressing reporter cell line generated in-house enabled the development of a luminescence-based assay to monitor and study BE-eVLP transduction. Transduction with BE-eVLPs exhibited sigmoidal kinetics, with logarithmic step increase in luminescence during the first 30 min, consistent with rapid VSV-G-mediated endocytosis,^23^^,^^30^^,^^31^ followed by gradual rise until plateauing at approximately 2.5–3 h post-transduction. With ∼30,000 cells, a step-change in luminescence was observed between 1 and 5 μL BE-eVLP doses. Much smaller, if not negligible, changes in luminescence were detected between 5- and 20-μL doses, revealing a ceiling in transduction that was reflected in levels of editing. In contrast to the proportional increase in both luminescence and editing observed when single doses of 1 or 2 μL of BE-eVLP preparation were applied to the LgBiT-expressing reporter cells, two sequential 1-μL doses, at time t = 0 and ∼3 h, produced no additional luminescence or editing compared to a single 1-μL dose. This suggests that early cellular responses to the first BE-eVLP dose could inhibit further uptake by endocytosis and/or exit from the endosome. It is conceivable that virus components in the BE-eVLPs serve as pathogen-associated molecular patterns that, recognized by pattern recognition receptors, trigger an antiviral state that redirects intracellular membrane and vesicular trafficking to lysosomes.^32^^,^^33^^,^^34^ Any BE-eVLPs within redirected endosomes may be degraded rather than released into the cytosol, thus hindering association between HiBiT and LgBiT.

Finally, two critical controls validated assay specificity and confirmed that luminescence signals from the LgBiT reporter cell line represent the endosomal route of BE-eVLP entry. Treatment with DrkBiT, which inhibits extracellular LgBiT complementation with HiBiT, produced no change in RLUs, verifying that luminescence specifically derives from intracellular complementation. Bafilomycin A1, which inhibits endosomal acidification, reduced luminescence, confirming that signals specifically reflect endosomal acidification-dependent transduction.

By proposing and evaluating new approaches for the quantification of VLPs, our study aims to contribute to robust titration of BE-eVLPs for normalization across experiments and laboratories. By introducing and validating a reporter assay to monitor transduction, this work seeks to facilitate future investigations into the mechanisms of BE-eVLP-mediated delivery and the identification of bottlenecks between delivery and execution of editing, particularly in the context of *in vivo* applications, when BE-eVLP-induced immune and cellular stress responses must be considered. The LgBiT reporter cell line can facilitate further design and refinement of eVLPs by enabling comparison of transduction and editing achieved by novel eVLP platforms and pseudotypes.

## Materials and methods

### Plasmids

Plasmids used in this study for eVLP production include pCMV-MMLVgag-3xNES-ABE8e-NG (Addgene plasmid #181754), a kind gift from David Liu; pBS-CMV-gagpol (Addgene plasmid #35614), a kind gift from Patrick Salmon; pCMV-VSV-G (Addgene plasmid #8454), a kind gift from Bob Weinberg; pCMV-VSV-G-HiBiT (Addgene plasmid #162594), a kind gift from Masaharu Somiya; and pU6-HEK2-sgRNA-HEAT, pU6-HEK3-sgRNA-HEAT, and pU6-CFex11-sgRNA-HEAT, produced by golden gate cloning^35^ of the 20-nucleotide (nt)-long spacer sequence (Table 2) into gRNA Cloning Vector Bbs I v.2 (Addgene plasmid #85586), a kind gift from Hodaka Fujii.

Plasmids used in this study for the development of the LgBiT HEK293 cell line include pAAVS1-LgBiT-Puro HDR donor plasmid, constructed from the LgBiT expression vector (Promega, Cat# N2681), and pAAVS1-SA-2A-Puro (Addgene plasmid #22075), a kind gift from Rudolf Jaenisch; sgRNA cloning vector (Addgene plasmid #85586), a kind gift from Hodaka Fujii; and Cas9 (Addgene plasmid #138557), a kind gift from Hyongbum Kim.

All the plasmids in this study were propagated in NEB 5-alpha chemically competent *E. coli* (NEB, Cat#C2987H) and purified for transfection using the PlasmidPlus Midi Kit (QIAGEN, Cat#12941) with endotoxin removal. Plasmid identity was confirmed by Sanger sequencing (Eurofins Genomics) or whole-plasmid sequencing with Oxford Nanopore Technologies (PlasmidSaurus) and pairwise alignment on Geneious Prime software platform.

### Cell culture

Gesicle Producer 293T cells (Takara Bio, Cat#632617), human embryonic kidney 293T (HEK293T) cells, and LgBiT (HEK293) cells (developed in-house as described below) were cultured in Dulbecco’s modified Eagle medium with GlutaMax (Thermo Fisher Scientific, Cat#10566024) supplemented with 10% fetal bovine serum (Merck, Cat#F7524) at 37°C with 5% CO_2_.

### Generation of the LgBiT cell line: Donor plasmid construction

A pAAVS1-LgBiT-Puro HDR donor plasmid was constructed from the LgBiT expression vector (Promega, Cat# N2681) and pAAVS1-SA-2A-Puro (Addgene plasmid #22075). First, the SalI restriction enzyme site in the LgBiT expression vector was mutated by site-directed mutagenesis (SDM) to prevent digestion in subsequent steps; SDM was performed using the Q5 Site-Directed Mutagenesis Kit (NEB, Cat#E0554S) and the SalI_SDM_fw and SalI_SDM_rv primers (Table 1). The resulting plasmid was then used as a template to amplify the CAG promoter and LgBiT fragment with primers CAG_SalI_fw and pA_SalI_rv containing SalI restriction sites added to their 5′ end (underlined in Table 1). The resulting PCR product (insert) and pAAVS1-SA-2A-Puro (vector) were digested at 37°C for 4 h with SalI-HF (NEB, Cat#R3138S). After digestion, pAAVS1-SA-2A-Puro was dephosphorylated with Antarctic phosphatase, (NEB, Cat#M0289) for 30 min at 37°C to prevent re-ligation. Digested products were run on an agarose gel, excised, and purified using the NEB, Cat#T1020). Ligation was performed with T4 DNA ligase (NEB, Cat#M0202S) using a 3:1 insert:vector molar ratio for 1 h at room temperature, followed by transformation of NEB 5-alpha competent *E. coli* and purification of the resulting pAAVS1-LgBiT-Puro donor plasmid.

**Table 1:** List of primers

Primer name	Sequence (5′-3′)
**LgBiT cell line generation**	

SalI_SDM_fw	ATAACGTCGATGTATTGCGGC
SalI_SDM_rv	CAGCTGACTTCGTACGAG
CAG_SalI_fw	CACTCAGTCGACAATATGACCGCCATGTTGG
pA_SalI_rv	ATTGTAGTCGACCGCCTCAGAAGGTACCTAAC

**LgBiT cell line PCR genotyping**	

AAVS1_fw	TCCTGAGTCCGGACCACTTT
AAVS1_rv	AGGATCCTCTCTGGCTCCAT
LgBiT_geno_fw	ACAGACAGCCGCCTACAAC

**Genome editing verification**	

HEK2_fw	CCAGCCCCATCTGTCAAACT
HEK2_rv	TGAATGGATTCCTTGGAAACAATGA
HEK3_fw	ATGTGGGCTGCCTAGAAAGG
HEK3_rv	CCCAGCCAAACTTGTCAACC
CFex11_fw	TCCAGACTTCACTTCTAATGGTG^11^
CFex11_rv	CTAACCGATTGAATATGGAGCCAA^11^

**sgRNA detection**	

qPCR_sgRNA_fw	TGCTGGAAACAGCATAGCAAGTTT
qPCR_sgRNA_rv	GACTCGGTGCCACTTTTTCAAGTT

**Plasmid DNA detection PCR and qPCR**	

AmpR_fw	TCCGGTTCCCAACGATCAAG
AmpR_rv	ACCCAGAAACGCTGGTGAAA
qPCR_sgRNA_fw	TGCTGGAAACAGCATAGCAAGTTT
qPCR_sgRNA_rv	GACTCGGTGCCACTTTTTCAAGTT
qPCR_VSVG_fw	CGAGCTTGTAGAAGGTTGGT
qPCR_VSVG_rv	AGATGGATACCAACTCGGAG
qPCR_gagpol_fw	CTACCAAGAACAACTGGACC
qPCR_gagpol_rv	TCAGCAGGACTGTGTAAGGT
qPCR_ABE_fw	CTTCCTGGTGGAAGAGGATA
qPCR_ABE_rv	TTCTCAGGTGGTAGATGGTG
qPCR_RPLP0_fw	GCAGCATCTACAACCCTGAAG
qPCR_RPLP0_rv	CACTGGCAACATTGCGGAC

### Generation of the LgBiT cell line: Electroporation

An sgRNA targeting the AAVS1 locus was made by cloning a 20-nt-long spacer sequence into an sgRNA cloning vector (Addgene plasmid #85586) by golden gate cloning. HEK293 cells were electroporated with 250 ng sgRNA, 250 ng Cas9 (Addgene plasmid #138557), and 500 ng pAAVS1-LgBiT-Puro plasmids using the Neon Transfection System 10 μL kit (Thermo Fisher, Cat#MPK1096). For each reaction, plasmid DNA was diluted up to 6 μL in BufferR and 200,000 cells were resuspended in 6 μL of BufferR. Diluted plasmids and cells were mixed and electroporated under the following conditions: 1250 V, 20 ms, 2 pulses. After electroporation, cells were transferred to a 24-well plate containing maintenance medium DMEM (Gibco, Cat#11965092) supplemented with 10% FBS and 1 μM AZD7648 (SelleckChem, Cat#S8843).

### Generation of the LgBiT cell line: Limiting dilution cloning

Three days post-electroporation, cells were dissociated and seeded in a limiting dilution assay to obtain a monoclonal cell line. Cells were diluted to 20,000 cells/well in maintenance medium and seeded in the first well of a 96-well plate. Serial dilutions (2-fold) were made first vertically along the first column and then horizontally. After 7 days, plates were scanned to identify wells growing a single colony. After ∼14 days, colonies were transferred to 24-well plates and expanded. A sample of the colony was taken for genotyping.

### Generation of the LgBiT cell line: PCR genotyping

Cell DNA was extracted from single colonies in 50 μL of QuickExtract DNA Extraction Solution (Lucigen, Cat#LGCQE09050) at 65°C for 15 min and 95°C for 5 min. PCR with AAVS1- and LgBiT-specific primers (Table 1) was performed using Q5 Hot Start High-Fidelity 2X Master Mix (NEB, Cat#M0492L) and carried out as follows: 98°C for 2 min, 30 cycles of 98°C for 10 s, 62°C for 30 s, and 72°C for 30 s, followed by a final 72°C extension for 2 min. PCR products were purified with Sera-Mag SpeedBeads Carboxyl Magnetic Beads (Cytiva, Cat#65152105050250).

### BE-eVLP production and purification

eVLPs used in this study were v.v4, produced as described by Banskota and colleagues.^1^ In brief, the vectors were produced by transient transfection of Gesicle Producer 293T cells (Takara Bio, Cat#632617) under 10 mL of medium in T-75 flasks (Sarstedt, Cat#83.3911.002) by jetPRIME reagent (Polyplus, Cat#101000001) according to the manufacturer’s instructions. For the production of non-HiBiT BE-eVLPs, we co-transfected pCMV-MMLVgag-3xNES-ABE8e-NG (1,125 ng), pBS-CMV-gagpol (3,375 ng), pCMV-VSV-G (400 ng) and target-specific pU6-sgRNA-HEAT (4,400 ng). For the production of HiBiT BE-eVLPs, we co-transfected pCMV-MMLVgag-3xNES-ABE8e-NG (1,125 ng), pBS-CMV-gagpol (3,375 ng), pCMV-VSV-G-HiBiT (400 ng), and target-specific pU6-sgRNA-HEAT (4,400 ng). For the production of non-HiBiT non-ABE eVLPs, we co-transfected pBS-CMV-gagpol (3,375 ng), pCMV-VSV-G (400 ng), and target-specific pU6-sgRNA-HEAT (4,400 ng). Approximately ∼40–48 h post-transfection, Gesicle cell supernatant was harvested and centrifuged for 5 min at 500 × *g* to remove cell debris. The clarified supernatant was filtered through a 0.45-mm PVDF filter. PEG precipitated eVLPs were concentrated ∼100-fold using PEG-it Virus Precipitation Solution (System Biosciences, Cat#LV825A-1) according to the manufacturer’s protocol. These were recovered as a pellet by centrifugation for 30 min at 1500 × *g* and 4°C and resuspended in the appropriate volume of cold PBS for the concentration required (e.g., 100 μL for one T75 flask of producer cell supernatant). Ultracentrifugation was carried out in 13.2-mL tubes (Beckman Coulter, Cat#344059), using an SW 41 Ti swinging-bucket rotor (Beckman Coulter, Cat#331362) in an Optima L-90K Ultracentrifuge (Beckman Coulter). eVLPs were pelleted through a 1.5-mL cushion of 20% (w/v) sucrose in PBS, at 26,000 rpm for 2 h at 4°C. Following ultracentrifugation, eVLP pellets were resuspended in PBS and centrifuged at 1,000 × *g* for 5 min at 4°C to remove debris. Replicates of eVLP preparations, for each target locus, were prepared using separate plasmid batches. For direct comparison of PEG precipitated vs. ultracentrifuged eVLPs (e.g., Figure 4), preparations were prepared in parallel (i.e., for each replicate, producer cells were transfected at the same time with the same plasmid batches, but clarified supernatant was either PEG precipitated or ultracentrifuged).

### RNA extraction from BE-eVLPs and DNase treatments

RNA was extracted from eVLPs using the QIAmp Viral RNA Mini Kit (QIAGEN, Cat#52904). A DNase I treatment step was included: DNase I and DNase buffer from QIAGEN RNase-Free DNase Set (QIAGEN, Cat#79254) were applied, according to the manufacturer’s instructions, on the membrane of the QIAmp Kit extraction columns during the RNA extraction workflow. Samples were incubated with DNase I for 15 min before proceeding with the final washes and elution. Extracted RNA was eluted in nuclease-free water (NFW). For whole-prep DNase treatment, we selected TURBO DNA-free Kit (Thermo Fisher, Cat#AM1907) with DNase inactivation reagent, according to the manufacturer’s rigorous DNase treatment protocol.

### Nucleic acid quantification

Plasmid DNA was quantified using the Qubit 1X dsDNA BR Assay Kit (Thermo Fisher, Cat#Q33266). RNA was quantified using the Qubit RNA HS Assay Kit (Thermo Fisher, Cat#Q32852). qPCR standard oligonucleotides were quantified using the Qubit 1X ssDNA Assay Kit (Thermo Fisher, Cat#Q10212).

### cDNA synthesis

100 ng of HiBiT or non-HiBiT BE-eVLP RNA were reverse transcribed with random hexamers using LunaScript RT SuperMix Kit (NEB, Cat#E3010S) for first-strand cDNA synthesis or added to no-RT controls, according to the manufacturer’s protocols.

### qPCR for quantification of sgRNA copy number in BE-eVLP preparations

qPCR for the quantification of sgRNA copy number was performed using the LightCycler 480_1536 Real-Time PCR Detection System (Roche) with LightCycler 480 SYBR Green I Master (Roche, Cat#04707516001). A chemically synthesized DNA oligonucleotide (Integrated DNA Technologies), corresponding to the cDNA sequence of HEK2, HEK3, or CFex11 sgRNA (qPCR_HEK2std, qPCR_HEK3std, or qPCR_CFex11std, Table 2), was used to generate a standard curve for quantification. The standard oligonucleotides (100 μM) were serially diluted 10-fold down to 10^−10^. The 100-fold dilution (10^−2^) of each oligonucleotide was quantified, and the concentration (ng/μL) of subsequent dilutions determined by inferring a 10-fold decrease. A 10 μL reaction volume was prepared, with 5 μL of SYBR Green I Master mix (Roche, Cat#04707516001), 0.5 μL of both forward and reverse primers (qPCR_sgRNA_fw and qPCR_sgRNA_rv, 10 μM, Table 1), 1.5 μL of NFW, and 2.5 μL of normalized cDNA sample (dilute 10- or 100-fold), or no-RT control, standard oligo, or NFW for no-template controls. Cycling settings included initial denaturation at 95°C for 5 min and 40 amplification cycles at 95°C for 10 s, 60°C for 20 s, and 72°C for 10 s, followed by a melt-curve analysis performed at 95°C for 5 s, 65°C for 1 min and continuous acquisition with a temperature gradient of 0.1°C/s. The standard curve was generated each time the samples were quantified, and all samples, controls, and standards were run in triplicate; outliers were discarded from replicates if the standard deviation of their Ct values exceeded 0.5. qPCR data were analyzed for quantification of sgRNA copy number per μL of BE-eVLP preparation as described below and outlined in the supplementary Excel template sheet.

**Table 2:** List of spacers and standard oligonucleotides

**sgRNA spacer name**	**Sequence (5′-3′)**
AAVS1	GGGGCCACTAGGGACAGGAT
HEK2	GAACACAAAGCATAGACTGC
HEK3	GGCCCAGACTGAGCACGTGA
CFex11	CAAAGCATGCCAACTAGAAG^11^
**Standard oligonucleotide name**	**Sequence (5′-3′)**
qPCR_HEK2std	AAAAAAAGCACCGACTCGGTGCC ACTTTTTCAAGTTGATAACGGAC TAGCCTTATTTAAACTTGCTATG CTGTTTCCAGCATAGCTCTTAAA CGCAGTCTATGCTTTGTGTTC
qPCR_HEK3std	AAAAAAAGCACCGACTCGG TGCCACTTTTTCAAGTTGAT AACGGACTAGCCTTATTTAA ACTTGCTATGCTGTTTCCAG CATAGCTCTTAAACTCACGT GCTCAGTCTGGGCC
qPCR_CFex11std	AAAAAAAGCACCGACTCGGT GCCACTTTTTCAAGTTGATAA CGGACTAGCCTTATTTAAACT TGCTATGCTGTTTCCAGCATA GCTCTTAAACCTTCTAGTTGG CATGCTTTG

### Quantification of sgRNA copy number per μL of BE-eVLP preparation

Equation 2 was applied to calculate the number of sgRNA copies per μL of qPCR reaction. In Equation 2, ng of test sample is the antilog of the log10 of the test sample extrapolated from the standard curve (Equation 1) and adjusted for the dilution of cDNA used in the reaction (100-fold), 6.022 × 10^23^ molecules/mol is Avogadro’s number, amplicon length (bp) is 69, 330 g/mol is the average molecular weight of 1 nt of ssDNA, and 1 × 10^9^ ng/g is the conversion factor for nanograms to grams. Next, to calculate the number of sgRNA copies per μL of BE-eVLP prep, (1) the copy number of sgRNAs per μL of qPCR reaction was multiplied by the cDNA dilution factor (10 or 100) and by the volume of the cDNA synthesis reaction (20 μL), (2) the derived value was divided by the volume of BE-eVLP RNA used in the cDNA synthesis reaction (sample-specific for normalization to 100 ng), and (3) this value was multiplied by the elution volume of the RNA extraction (e.g., 30 μL) and (4) divided by the initial volume of BE-eVLP preparation used for RNA extraction (e.g., 5 μL).

### PCR for the detection of plasmid DNA in BE-eVLP preparations or transduced cells

For the detection of the Amp^R^ cassette, a 10 μL reaction volume was prepared, with 5 μL of SYBR Green I Master mix (Roche, Cat#04707516001), 0.5 μL of both forward and reverse primers (AmpR_fw and AmpR_rv, 10 μM), 1.5 μL of NFW, and 2.5 μL of normalized BE-eVLP cDNA sample, or no-RT control, sgRNA-encoding plasmid (100 ng), or NFW for no-template control. Cycling settings included initial denaturation at 95°C for 5 min and 40 amplification cycles at 95°C for 10 s, 60°C for 20 s, and 72°C for 10 s.

### qPCR for the detection of plasmid DNA in transduced cells

qPCR was performed using the LightCycler 480_1536 Real-Time PCR Detection System (Roche) with LightCycler 480 SYBR Green I Master (Roche, Cat#04707516001). A standard curve was generated for each plasmid used in the production of the BE-eVLPs. For each target, a 10 μL reaction volume was prepared, with 5 μL of SYBR Green I Master mix (Roche, Cat#04707516001), 0.5 μL of both forward and reverse primers (10 μM, Table 1), 1.5 μL of NFW, and 2.5 μL of DNA from transduced cells (2 or 7 days post-transduction), plasmid, or NFW for no-template controls. To normalize against genome copy number across all conditions, primers were included for *RPLP0* (Table 1), as an internal control gene. Cycling settings included initial denaturation at 95°C for 5 min and 40 amplification cycles at 95°C for 10 s, 60°C for 20 s, 72°C for 10 s, followed by a melt-curve analysis performed at 95°C for 5 s, 65°C for 1 min, and continuous acquisition with a temperature gradient of 0.1°C/s.

### Lytic luminescence HiBiT assay

The Nano-Glo HiBiT Lytic Detection System (Promega, Cat#N3030) was used for the quantification of VSV-G-HiBiT abundance in BE-eVLP preparations according to the manufacturer’s instructions. Briefly, eVLPs were quantified undiluted or diluted 10- or 100-fold in PBS; 1 μL of each eVLP preparation (or PBS, or conditioned medium) was mixed with 49 μL of lytic buffer, 1 μL of lytic substrate (furimazine), 0.5 μL of LgBiT protein, and 49 μL of PBS, to a final volume of 100 μL. Samples were prepared in an opaque white plate with clear lid (Corning, Cat # 10337461) and incubated for 10 min at room temperature on an orbital shaker prior to luminescence reading. Luminescence was detected with the plate reader CLARIOstar Plus (BMG LABTECH), using top optic, at 470-80 emission, 1 kinetic window, 1 interval, 0.8 s interval time, and no shaking.

### BE-eVLP transduction of LgBiT HEK293 cell cultures

Approximately 24 h prior to transduction, ∼25–30,000 LgBiT HEK293 cells were seeded into each well of a 96-well Clear Bottom TC Surface Microplate (Thermo Fisher Scientific, Cat#165306) and cultured in Dulbecco’s modified Eagle medium with GlutaMax (Thermo Fisher Scientific, Cat#10566024) supplemented with 10% fetal bovine serum (Merck, Cat#F7524) at 37°C with 5% CO_2_. On the day of transduction, conditioned medium was replaced with fresh medium supplemented with 10% fetal bovine serum added to each well to a final volume of 100 μL, together with 1X Nano-Glo Vivazine substrate (Promega, Cat#N2580) and required volumes of eVLPs or PBS. Cells were incubated for ∼48–72 h with no medium change, prior to extraction of genomic DNA.

### DrkBiT and bafilomycin treatment of LgBiT HEK293 cell cultures

Prior to transduction, designated LgBiT HEK293 cell monolayers were incubated for 1 h with 1X DrkBiT Peptide solution (Promega, Cat#CS3002A02) according to the manufacturer’s instructions, Bafilomycin A1 (100 nM, Merck, Cat#B1793-2UG), or equivalent volume of DMSO.

### Live-cell luminescence LgBiT assay

On the day of transduction, luminescence was recorded using the plate reader CLARIOstar Plus (BMG Labtech), at 37°C, with top optic, at 470-80 nm emission, 1 kinetic window, 1 interval, 0.8 s interval time, and no shaking. Luminescence was recorded prior to the experiment (e.g., time −1 h), immediately after transduction (time t = 0 h), and at defined intervals post-transduction. Luminescence readings at each time point were divided by pre-transduction RLUs to ensure per-well normalization.

### Total cell DNA extraction and amplification

Total cell DNA from transduced and mock-transduced LgBiT HEK293 cells was extracted 48–72 h post-transduction using 20 μL of QuickExtract DNA Extraction Solution according to the manufacturer’s instructions. Extracted DNA was diluted 5-fold in NFW for downstream PCR amplification. Target regions were amplified for editing verification using Q5 High-Fidelity 2X Master Mix (NEB, Cat#M0492L). A 25 μL reaction volume was prepared, with 12.5 μL of Q5 Master mix, 1.25 μL of both forward and reverse primers (10 μM, Table 1), 8 μL of NFW, and 2 μL of DNA, or NFW for no-template controls. Cycling settings included initial denaturation at 95°C for 3 min and 30 amplification cycles at 95°C for 15 s, 58°C for 20 s for HEK2 or 61°C for 20 s for HEK3, 72°C for 30 s, followed by final extension at 72°C for 1 min.

### Gene editing verification

PCR amplicons were purified with Sera-Mag SpeedBeads Carboxyl Magnetic Beads (Cytiva, Cat#65152105050250) and Sanger sequenced (Eurofins Genomics). Sanger sequencing.ab1 files were analyzed for editing verification and quantification using EditR software.

### Quantification, statistical analysis, and figure generation

No statistical methods were used to predetermine sample size. Statistical analyses (two-way ANOVA or multiple *t* tests) were performed using GraphPad Prism software. Data are shown as mean and standard deviation (SD), or mean and standard error of the mean (SEM), as indicated in each figure legend. Graphical abstract and Figures 1A, 1B, 2A, 3A, and S3A were generated using BioRender.

## Data and code availability

All the data and custom code generated in this study are available from the lead contact, Martina F. Scallan (m.scallan@ucc.ie), upon request. This study generated an LgBiT-expressing HEK293 cell line and used custom code for HiBiT-like motif search in R with assistance from Claude AI (Anthropic, Claude Sonnet 4.5).

## Acknowledgments

This research was supported by DRUMM22G0-COLLAB from the 10.13039/100000897Cystic Fibrosis Foundation and SRC020 from the 10.13039/501100000292Cystic Fibrosis Trust and the 10.13039/100000897Cystic Fibrosis Foundation. The authors would like to thank Karen R. Dunn for her contribution to the conceptualization of the HiBiT assay, and James P. Costello, Michael Carroll, and Patrick Hand for their preliminary work on the development of the RT-qPCR assay.

## Author contributions

Conceptualization, L.N., J.B.M., and M.F.S.; initial developmental work on RT-qPCR for BE-eVLP quantification, L.N. and L.L.; BE-eVLP production and quantification, L.N.; cell line design and generation, E.C.; transduction experiments and downstream assays and analysis, L.N.; tissue culturing, L.N. and E.C.; data curation and formal analysis, L.N.; supervision, M.F.S.; writing – original draft, L.N.; writing – review & editing, L.N., P.T.H., and M.F.S.; funding acquisition, P.T.H.; resources, M.F.S. and P.T.H.

## Declaration of interests

The authors declare no competing interests.

## Declaration of generative AI and AI-assisted technologies in the writing process

During the preparation of this work the authors used Claude AI (Anthropic, Claude Sonnet 4.5) in order to generate custom code for HiBiT-like motif search in R. After using this tool, the authors reviewed and edited the content as needed and take full responsibility for the content of the published article.

## Supplemental information

Document S1. Figures S1–S5 and Tables S2 and S3

Table S1. Excel template sheet for RT-qPCR analysis

Document S2. Article plus supplemental information

## References

[1] Banskota S., Raguram A., Suh S., Du S.W., Davis J.R., Choi E.H., Wang X., Nielsen S.C., Newby G.A., Randolph P.B. (2022). Engineered virus-like particles for efficient in vivo delivery of therapeutic proteins. Cell.

[2] An M., Raguram A., Du S.W., Banskota S., Davis J.R., Newby G.A., Chen P.Z., Palczewski K., Liu D.R. (2024). Engineered virus-like particles for transient delivery of prime editor ribonucleoprotein complexes in vivo. Nat. Biotechnol..

[3] Raguram A., An M., Chen P.Z., Liu D.R. (2025). Directed evolution of engineered virus-like particles with improved production and transduction efficiencies. Nat. Biotechnol..

[4] Nicosia L., Pranke I., Latorre R.V., Murray J.B., Lonetti L., Cavusoglu-Doran K., Dreano E., Costello J.P., Carroll M., Melotti P. (2025). Adenine base editing with engineered virus-like particles rescues the CFTR mutation G542X in patient-derived intestinal organoids. iScience.

[5] Hwang H.Y., Lee M., Yi H., Seok C., Lim K., Na Y.R., Kang J.S., Park J.H., Kim D. (2025). Engineered Sdd7 cytosine base editors with enhanced specificity. Nat. Commun..

[6] Du S.W., Palczewska G., Dong Z., Lauterborn J.C., Kaipa B.R., Yan A.L., Hołubowicz R., Ha S., Chen P.Z., Gall C.M. (2025). TIGER: A tdTomato in vivo genome-editing reporter mouse for investigating precision-editor delivery approaches. Proc. Natl. Acad. Sci. USA.

[7] Renner T.M., Tang V.A., Burger D., Langlois M.-A. (2020). Intact Viral Particle Counts Measured by Flow Virometry Provide Insight into the Infectivity and Genome Packaging Efficiency of Moloney Murine Leukemia Virus. J. Virol..

[8] Farley D.C., Iqball S., Smith J.C., Miskin J.E., Kingsman S.M., Mitrophanous K.A. (2007). Factors that influence VSV-G pseudotyping and transduction efficiency of lentiviral vectors - In vitro and in vivo implications. J. Gene Med..

[9] Kaczmarczyk S.J., Sitaraman K., Young H.A., Hughes S.H., Chatterjee D.K. (2011). Protein delivery using engineered virus-like particles. Proc. Natl. Acad. Sci. USA.

[10] Richter M.F., Zhao K.T., Eton E., Lapinaite A., Newby G.A., Thuronyi B.W., Wilson C., Koblan L.W., Zeng J., Bauer D.E. (2020). Phage-assisted evolution of an adenine base editor with improved Cas domain compatibility and activity. Nat. Biotechnol..

[11] Sousa A.A., Hemez C., Lei L., Traore S., Kulhankova K., Newby G.A., Doman J.L., Oye K., Pandey S., Karp P.H. (2024). Systematic optimization of prime editing for the efficient functional correction of CFTR F508del in human airway epithelial cells. Nat. Biomed. Eng..

[12] Komor A.C., Kim Y.B., Packer M.S., Zuris J.A., Liu D.R. (2016). Programmable editing of a target base in genomic DNA without double-stranded DNA cleavage. Nature.

[13] Gosalia N., Harris A. (2015). Chromatin dynamics in the regulation of CFTR expression. Genes.

[14] Riesenberg S., Helmbrecht N., Kanis P., Maricic T., Pääbo S. (2022). Improved gRNA secondary structures allow editing of target sites resistant to CRISPR-Cas9 cleavage. Nat. Commun..

[15] Chen B., Gilbert L.A., Cimini B.A., Schnitzbauer J., Zhang W., Li G.W., Park J., Blackburn E.H., Weissman J.S., Qi L.S., Huang B. (2013). Dynamic imaging of genomic loci in living human cells by an optimized CRISPR/Cas system. Cell.

[16] Jinek M., Chylinski K., Fonfara I., Hauer M., Doudna J.A., Charpentier E. (2012). A programmable dual-RNA-guided DNA endonuclease in adaptive bacterial immunity. Science.

[17] Ma H., Bell K.N., Loker R.N. (2021). qPCR and qRT-PCR analysis: Regulatory points to consider when conducting biodistribution and vector shedding studies. Mol. Ther. Methods Clin. Dev..

[18] Whelan J.A., Russell N.B., Whelan M.A. (2003). A method for the absolute quantification of cDNA using real-time PCR. J. Immunol. Methods.

[19] Fan F., Wood K.V. (2007). Bioluminescent assays for high-throughput screening. Assay Drug Dev. Technol..

[20] Hall M.P., Unch J., Binkowski B.F., Valley M.P., Butler B.L., Wood M.G., Otto P., Zimmerman K., Vidugiris G., MacHleidt T. (2012). Engineered luciferase reporter from a deep sea shrimp utilizing a novel imidazopyrazinone substrate. ACS Chem. Biol..

[21] Dixon A.S., Schwinn M.K., Hall M.P., Zimmerman K., Otto P., Lubben T.H., Butler B.L., Binkowski B.F., MacHleidt T., Kirkland T.A. (2016). NanoLuc Complementation Reporter Optimized for Accurate Measurement of Protein Interactions in Cells. ACS Chem. Biol..

[22] Schwinn M.K., Machleidt T., Zimmerman K., Eggers C.T., Dixon A.S., Hurst R., Hall M.P., Encell L.P., Binkowski B.F., Wood K.V. (2018). CRISPR-Mediated Tagging of Endogenous Proteins with a Luminescent Peptide. ACS Chem. Biol..

[23] Somiya M., Kuroda S. (2021). Real-Time Luminescence Assay for Cytoplasmic Cargo Delivery of Extracellular Vesicles. Anal. Chem..

[24] Yamamoto M., Du Q., Song J., Wang H., Watanabe A., Tanaka Y., Kawaguchi Y., Inoue J.I., Matsuda Z. (2019). Cell cell and virus cell fusion assay based analyses of alanine insertion mutants in the distal 9 portion of the JRFL gp41 subunit from HIV-1. J. Biol. Chem..

[25] Roberts P.C., Kipperman T., Compans R.W. (1999). Vesicular Stomatitis Virus G Protein Acquires pH-Independent Fusion Activity during Transport in a Polarized Endometrial Cell Line. J. Virol..

[26] Mauvezin C., Neufeld T.P. (2015). Bafilomycin A1 disrupts autophagic flux by inhibiting both V-ATPase-dependent acidification and Ca-P60A/SERCA-dependent autophagosome-lysosome fusion. Autophagy.

[27] Lu T., Zhu Z., Wu J., She H., Han R., Xu H., Qin Z.H. (2019). DRAM1 regulates autophagy and cell proliferation via inhibition of the phosphoinositide 3-kinase-Akt-mTOR-ribosomal protein S6 pathway. Cell Commun. Signal..

[28] Kim S., Kim D., Cho S.W., Kim J., Kim J.S. (2014). Highly efficient RNA-guided genome editing in human cells via delivery of purified Cas9 ribonucleoproteins. Genome Res..

[29] Tu Z., Yang W., Yan S., Yin A., Gao J., Liu X., Zheng Y., Zheng J., Li Z., Yang S. (2017). Promoting Cas9 degradation reduces mosaic mutations in non-human primate embryos. Sci. Rep..

[30] Le Blanc I., Luyet P.P., Pons V., Ferguson C., Emans N., Petiot A., Mayran N., Demaurex N., Fauré J., Sadoul R. (2005). Endosome-to-cytosol transport of viral nucleocapsids. Nat. Cell Biol..

[31] Johannsdottir H.K., Mancini R., Kartenbeck J., Amato L., Helenius A. (2009). Host Cell Factors and Functions Involved in Vesicular Stomatitis Virus Entry. J. Virol..

[32] Zepeda-Cervantes J., Ramírez-Jarquín J.O., Vaca L. (2020). Interaction Between Virus-Like Particles (VLPs) and Pattern Recognition Receptors (PRRs) From Dendritic Cells (DCs): Toward Better Engineering of VLPs. Front. Immunol..

[33] Muhammad I., Contes K., Bility M.T., Tang Q. (2025). Chasing Virus Replication and Infection: PAMP-PRR Interaction Drives Type I Interferon Production, Which in Turn Activates ISG Expression and ISGylation. Viruses.

[34] Spence J.S., He R., Hoffmann H.H., Das T., Thinon E., Rice C.M., Peng T., Chandran K., Hang H.C. (2019). IFITM3 directly engages and shuttles incoming virus particles to lysosomes. Nat. Chem. Biol..

[35] Engler C., Kandzia R., Marillonnet S. (2008). A one pot, one step, precision cloning method with high throughput capability. PLoS One.

