# Supplementary material for: RT-qPCR and split-luciferase assays enable batch standardization and analysis of engineered virus-like particle transduction
Source: Mol Ther Adv. 2026 Feb 9;34(1):201685. doi: 10.1016/j.omta.2026.201685 (PMC13148891; doi:10.1016/j.omta.2026.201685)
Supplement: Document S1. Figures S1–S5 and Tables S2 and S3 [file mmc1.pdf]

OMTA, Volume *34*

Supplemental information

RT-qPCR and split-luciferase assays enable batch standardization and analysis of engineered virus-like particle transduction

Lucia Nicosia, Joss B. Murray, Emma Collins, Lisa Lonetti, Patrick T. Harrison, and Martina F. Scallan

Supplemental Material

Table S1. Excel template sheet for RT-qPCR analysis.

Table S2. Results of computational search of top three HiBiT-like nucleotide sequences in BE-eVLP plasmids, with mismatches (MMs).

Plasmid	Rank	Frame	Strand	Start_Pos_AA	Start_Pos_DNA	Match_Sequence	MMs
pCMV-VSV-G-HiBiT						VSGWRLFKKIS	
pCMV-MMLVgag-3xNES-ABE8e-NG	1	1	F	1805	5413	VVGTALIKKYP	6
pCMV-MMLVgag-3xNES-ABE8e-NG	2	1	F	2529	7585	VSPWKLPRALS	6
pCMV-MMLVgag-3xNES-ABE8e-NG	3	1	F	3699	11095	NSGWRGNILIG	6
pBS-CMV-gagpol	1	1	F	721	2161	VSGEKQRKKKN	6
pBS-CMV-gagpol	2	2	F	686	2057	VREVRRFKKQD	6
pBS-CMV-gagpol	3	2	R	202	8720	VSPWKLPRALS	6
pCMV-VSV-G	1	3	F	1785	5355	VSGSRWLIRIS	5
pCMV-VSV-G	2	1	F	279	835	VSPWKLPRALS	6
pCMV-VSV-G	3	1	F	1449	4345	NSGWRGNILIG	6

Table S3. Results of computational search of top three HiBiT-like amino acid sequences in BE-eVLP plasmids, by property similarity, with mismatches (MMs).

Plasmid	Rank	Frame	Strand	Start_Pos_AA	Start_Pos_DNA	Match_Sequence	MMs
pCMV-VSV-G-HiBiT						VSGWRLFKKIS	
pCMV-MMLVgag-3xNES-ABE8e-NG	1	1	F	2529	7585	VSPWKLPRALS	2
pCMV-MMLVgag-3xNES-ABE8e-NG	2	3	R	276	10536	VNGAELLRHFG	2
pCMV-MMLVgag-3xNES-ABE8e-NG	3	1	F	1145	3433	LTLLKALVRQQ	3
pBS-CMV-gagpol	1	1	F	1699	5095	LRPAKLVHKST	2
pBS-CMV-gagpol	2	2	R	202	8720	VSPWKLPRALS	2
pBS-CMV-gagpol	3	3	R	2899	628	VNGAELLRHFG	2
pCMV-VSV-G	1	1	F	279	835	VSPWKLPRALS	2
pCMV-VSV-G	2	1	F	2000	5998	YCLFFLYHRVN	2
pCMV-VSV-G	3	2	R	907	3786	VNGAELLRHFG	2

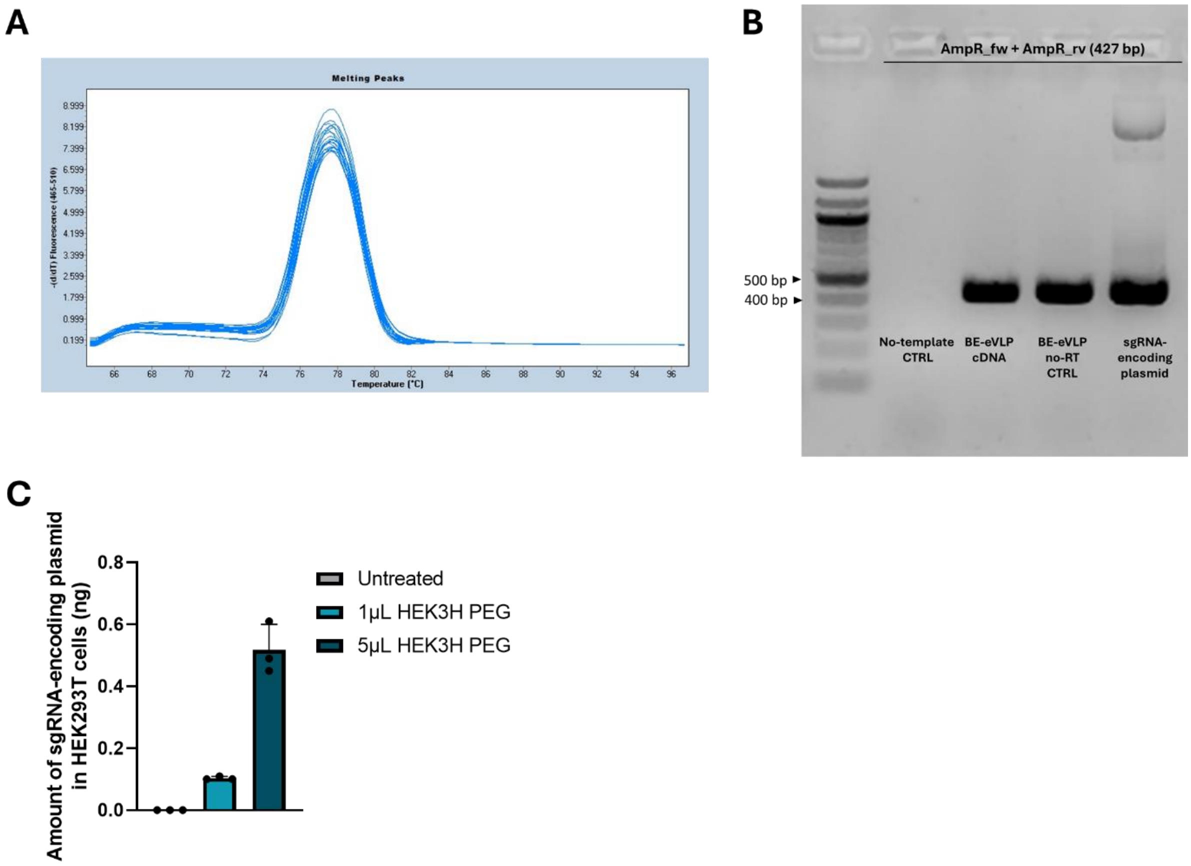

Figure S1. RT-qPCR assay specificity and plasmid DNA carryover.

(A) Representative qPCR melting peaks from amplified standard oligos. **(B)** Agarose gel electrophoresis of PCR amplicon product (427 bp) representing a region within the Ampicillin resistance cassette in (from left to right): no-template control, BE-eVLP cDNA, BE-eVLP no-RT control and sgRNA-encoding plasmid. **(C)** qPCR quantification of ng of sgRNA-encoding plasmid in ~30,000 HEK293T cells untreated or transduced with 1 or 5 µL of a PEG precipitated BE-eVLP preparation. n=3; bars represent mean ± SEM.

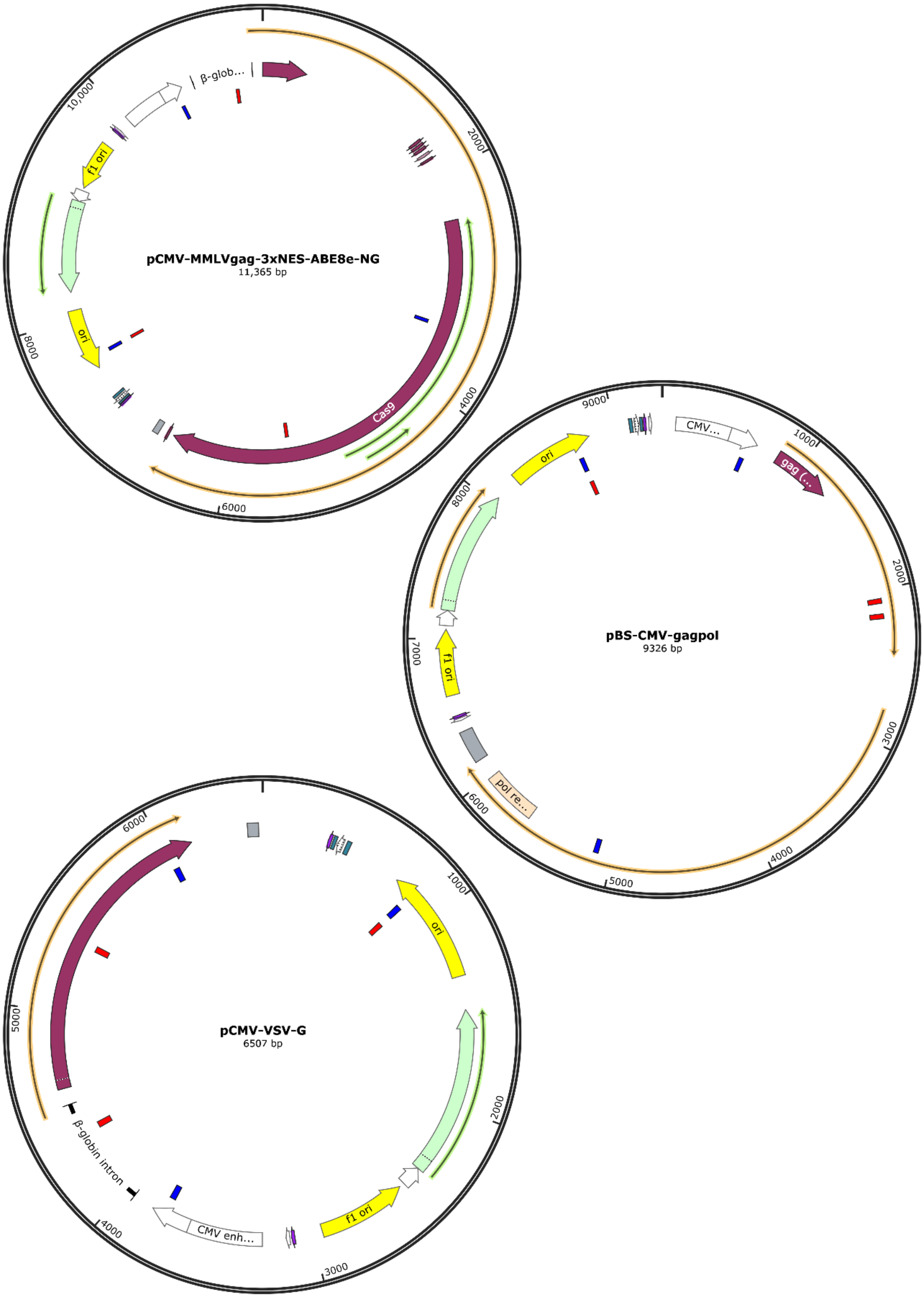

Figure S2. Annotated maps of BE-eVLP plasmids. Plasmid maps with top three HiBiT-like nucleotide sequences annotated in red and top three HiBiT-like amino acid sequences annotated in blue. Maps were generated on Snapgene.

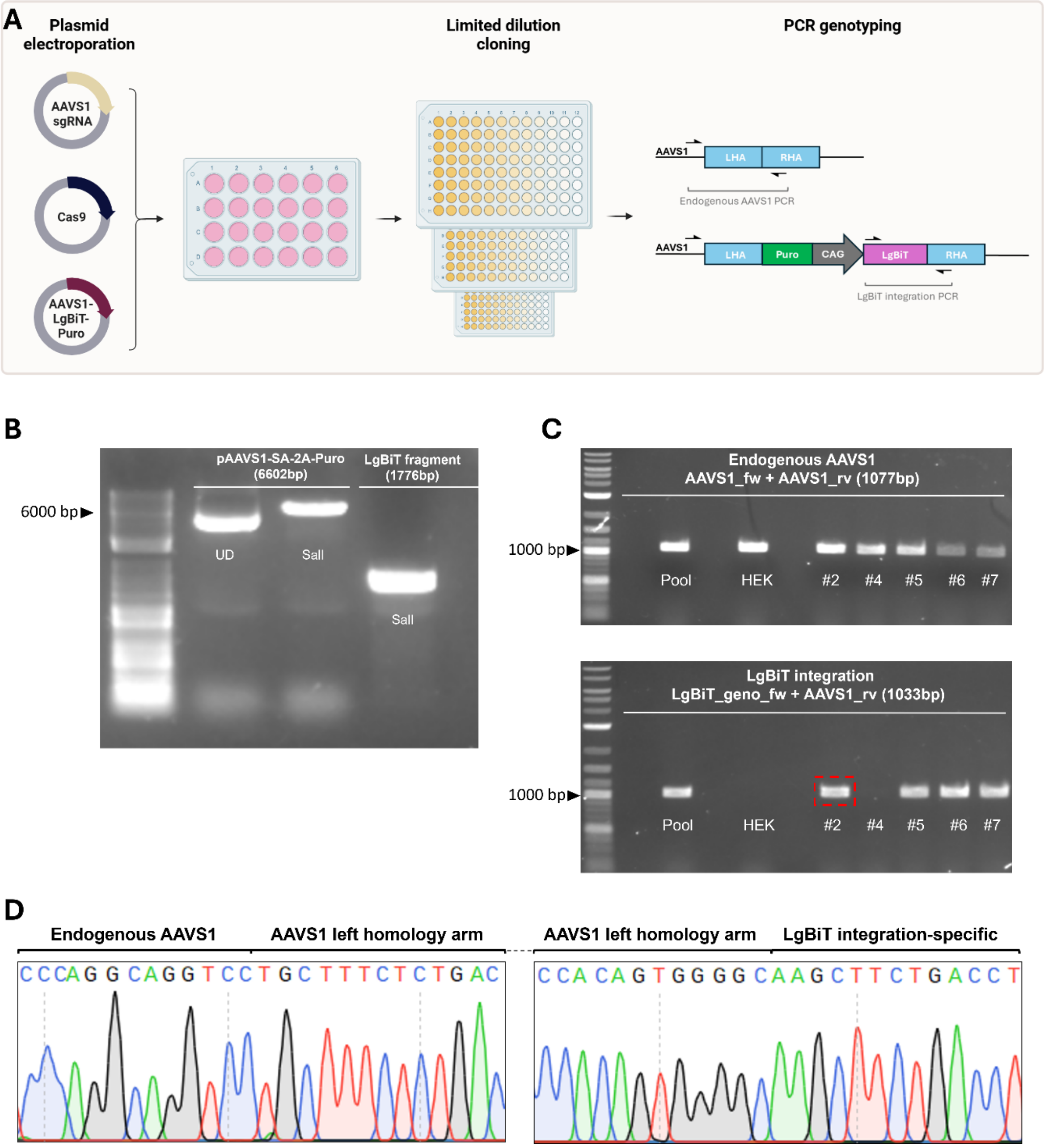

Figure S3. Generation of the LgBiT cell line in HEK293 cells.

(A) LgBiT cell line generation workflow. HEK293 cells were first electroporated with pAAVS1-LgBiT donor and Cas9/gRNA vectors, followed by limited dilution to obtain monoclonal cell lines. Clones were screened by genotyping PCRs. **(B)** pAAVS1-SA-2A-Puro and LgBiT PCR fragment digestion with SalI prior to ligation. **(C)** PCRs specific for the endogenous AAVS1 locus (upper) or LgBiT integration (lower) in clones (#2, 4-7). The pool of electroporated cells prior to dilution assay (pool) and untargeted HEK293 cells (HEK) respectively represent positive and negative PCR controls for integration of LgBiT. **(D)** Sanger sequencing chromatograms of LgBiT integration PCR amplicon from clone #2 confirm LgBiT integration into the AAVS1 locus.

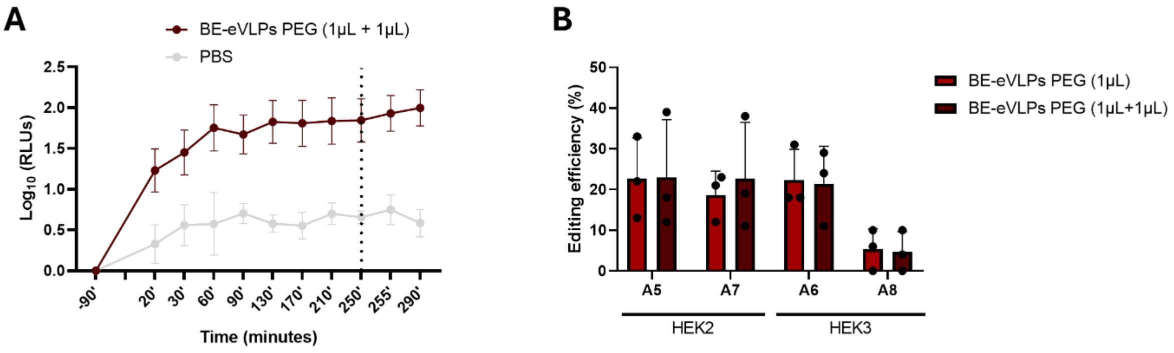

Figure S4. Sequential transduction with BE-eVLPs. (A) Luminescence readings (Log10 RLUs) of PBS and 1 µL of three independent PEG precipitated HiBiT-BE-eVLP preparations, monitored for ~3 hours prior to redosing with another 1 µL of the same preparations. Dotted line indicates the last luminescence reading prior to redosing. n=3; bars represent mean ± SD. **(B)** Editing efficiency (%) analyzed with EditR software at position A5 and A7 of the HEK2 protospacer, and A6 and A8 of the HEK3 protospacer, using 1 µL or 1 + 1 µL of three independent PEG precipitated BE-eVLP preparations; n=3; bars represent mean ± SD.

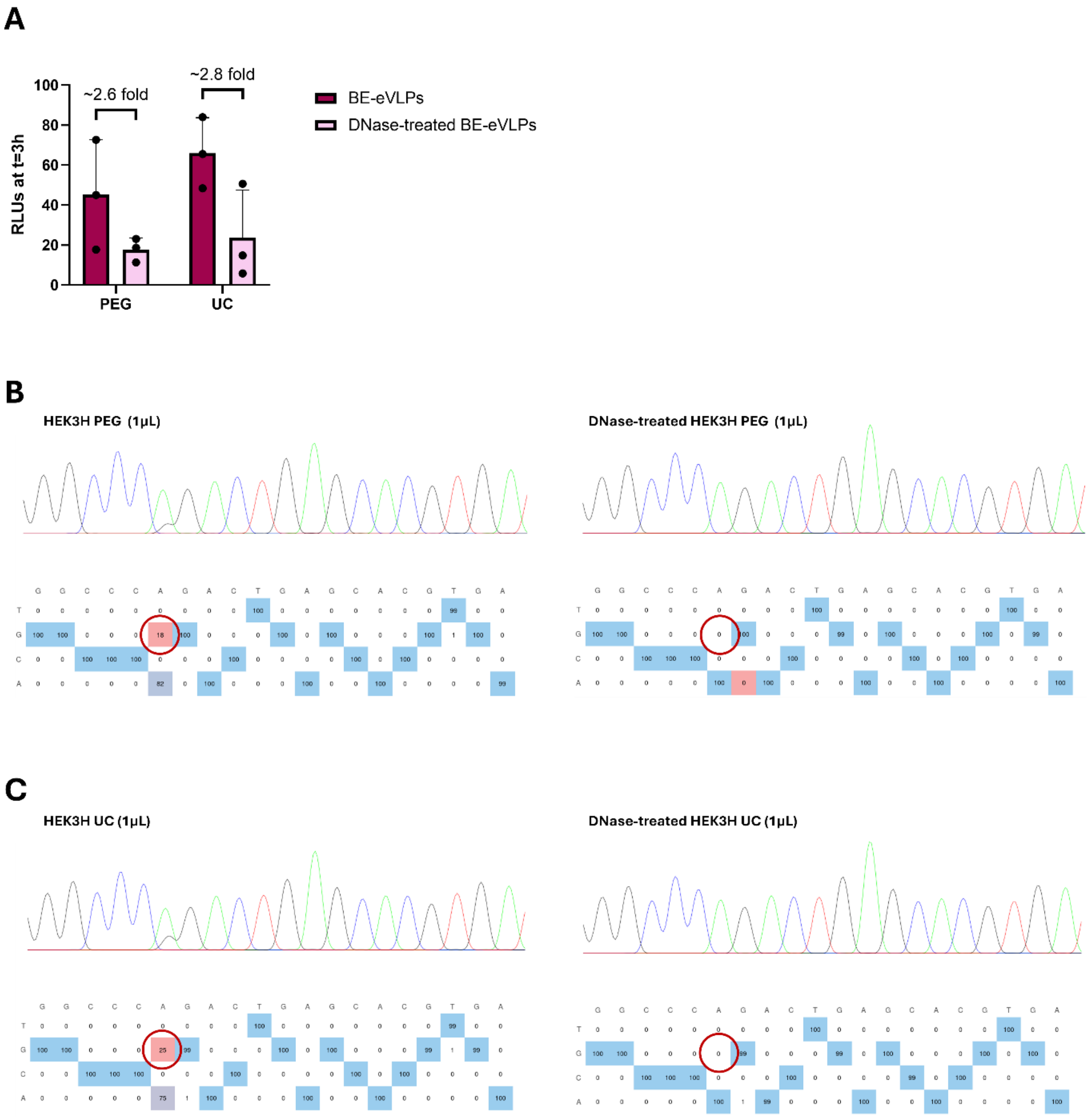

Figure S5. Transduction with DNase-treated BE-eVLPs. (A) Luminescence readings (RLUs) at time t=3h of three independent BE-eVLP preparations, that were either PEG precipitated or ultracentrifuged, with or without whole-prep DNase treatment. n=3; bars represent mean ± SD. **(B)** HEK3 locus editing efficiency (%) analyzed with EditR software, from cells transduced with 1 µL of a PEG precipitated BE-eVLP preparation, with (right) or without (left) DNase treatment. Target A6 is circled in red. **(C)** HEK3 locus editing efficiency (%) analyzed with EditR software, from cells transduced with 1 µL of an ultracentrifuged BE-eVLP preparation, with (right) or without (left) DNase treatment. Target A6 is circled in red.
